# Adiponectin/AdipoR Research and Its Implications for Lifestyle-Related Diseases

**DOI:** 10.3389/fcvm.2019.00116

**Published:** 2019-08-14

**Authors:** Masato Iwabu, Miki Okada-Iwabu, Toshimasa Yamauchi, Takashi Kadowaki

**Affiliations:** ^1^Department of Diabetes and Metabolic Diseases, Graduate School of Medicine, The University of Tokyo, Tokyo, Japan; ^2^Laboratory for Advanced Research on Pathophysiology of Metabolic Diseases, The University of Tokyo, Tokyo, Japan; ^3^Precursory Research for Embryonic Science and Technology (PRESTO), Japan Science and Technology Agency, Kawaguchi, Japan; ^4^AMED-CREST, Japan Agency for Medical Research and Development, Tokyo, Japan; ^5^Department of Prevention of Diabetes and Life-Style Related Diseases, The University of Tokyo, Tokyo, Japan; ^6^Department of Metabolism and Nutrition, Faculty of Medicine, Mizonokuchi Hospital, Teikyo University, Kawasaki, Japan

**Keywords:** adiponectin, AdipoR, AdipoRon, exercise-mimicking agents, lifestyle-related diseases

## Abstract

The number of patients with obesity continues to increase seriously worldwide. It has become clear that, against a background of insulin resistance, obesity induces the so-called metabolic syndrome consisting of diabetes, hypertension, and dyslipidemia, leading, consequently, to an increased incidence of cardiovascular disease in affected individuals. It is shown that environmental factors, e.g., high-fat diet and lack of physical activity, not only promote the onset of obesity but lead to impairment of the action of adiponectin and its receptors, thus accounting in part for the onset of insulin resistance, type 2 diabetes/metabolic syndrome, and atherosclerosis in modern society. This review is intended to highlight some milestones in adipocyte research from the discovery of the insulin-sensitizing properties of adiponectin to the elucidation of the structures of its receptors, as well as to clarify their therapeutic implications and prospects for lifestyle-related diseases.

## Role of Adipokines in Obesity-Associated Insulin Resistance

From the early 1990s onward, besides storing excess energy in the form of triglycerides, adipocytes have also been shown to function as endocrine organs secreting a variety of physiologically active substances currently known as “adipokines” (1–3). Again, while the mechanisms through which obesity induces insulin resistance remained largely unknown, it has now become clear that enlarged adipocytes secrete adipokines, such as TNF-α and MCP-1, in large amounts, thus not only impairing insulin signaling in the skeletal muscle and liver but inducing systemic insulin resistance (4, 5). In contrast, it is shown that the production/secretion of the “beneficial adipokine” adiponectin is decreased in obesity (6).

## Milestones in Adiponectin Research: Discovery of Adiponectin and Elucidation of Its Functions

Adiponectin has been originally identified through different approaches by four independent research groups as a protein secreted and expressed exclusively in adipose tissue (7–10). With a molecular weight of about 30 kDa, adiponectin is shown to consist of an amino-terminal signal sequence, a specific variable region containing cysteine residues essential for multimer formation of adiponectin, a collagen-like domain, and a carboxyl-terminal globular domain. Adiponectin has thus come to be classified by its structural characteristics as a member of the complement 1q family. Despite having no homology to TNF-α in the primary amino-acid sequence, adiponectin is shown to be homologous to TNF-α in structure (11). The adiponectin trimer represents the basic building block of adiponectin multimers and is shown to be formed through hydrophobic interactions within the globular domain and stabilized through interactions within the collagen-like domains in a triple helix structure. In contrast, adiponectin hexamers and high-molecular-weight multimers requires an intermolecular disulfide bond between the cysteine residues conserved within a specific variable region for their formation (12–14).

At the time of its discovery of adiponectin, its functions remained virtually unknown. To date, however, it is shown that while adiponectin is decreased, and insulin resistance and dyslipidemia are induced, in a mouse model of obesity/type 2 diabetes, physiological doses of adiponectin improve these conditions in this mouse model (15). It is also shown that globular adiponectin promotes fatty acid oxidation (16) and increases insulin sensitivity in the liver thus inhibiting hepatic gluconeogenesis and decreasing blood glucose levels (17, 18). These experimental results suggest that adiponectin is decreased in obesity leading to the onset of insulin resistance and the metabolic syndrome, while adiponectin supplementation represents an effective therapeutic option for the metabolic syndrome associated with obesity.

Again, analysis of adiponectin-deficient mice (19–21) and adiponectin transgenic mice (22, 23) has revealed the long-term action of adiponectin *in vivo*, where insulin resistance, impaired glucose tolerance, hypertension, and dyslipidemia were shown to be present in the former, while insulin resistance and diabetes were shown to be improved in the latter.

## Current Insight Into the Pathophysiological Role of Adiponectin in Obesity-Related Diseases

While adiponectin is shown to be present in the blood as varying multimers, it is shown to be available primarily as low-molecular-weight (LMW) trimers, middle-molecular-weight (MMW) hexamers, and high-molecular-weight (HMW) multimers (12, 13), with the HMV multimers being the most potent of all adiponectin multimers in activating AMP kinase (AMPK) (24, 25). It is also reported that adiponectin is regulated by transcription factors in adipose tissue, such as peroxisome proliferator-activated receptor-γ (PPAR-γ) (26), sterol regulatory element-binding protein 1c (SREBP1c) (27), and CCAAT-enhancer-binding protein-α (C/EBP-α) (28). Again, while thiazolidinediones (TZDs) are shown to upregulate the expression of adiponectin via PPAR-γ (26), the mechanisms through which TZDs promote the secretion of HMW adiponectin multimers include endoplasmic reticulum chaperones, such as ERp44 and Ero-1Lα (29). Interestingly, it is also reported that blood adiponectin levels are elevated during fasting or starvation or under caloric restriction, and the mechanisms involved include elevated blood levels of HMW adiponectin associated with the activation of sirtuin 1 (SIRT1) (30, 31), improvements in insulin resistance as shown in the liver of high-fat diet-fed mice (32), increased expression of adiponectin and inhibition of inflammatory cytokines (33) associated with the deacetylation by SIRT1 of Lus268 and Lys293 in PPAR-γ.

On the other hand, adiponectin is found to be decreased in obesity, with its blood level shown to be inversely correlated with insulin resistance in humans (6). The influence of insulin on the blood level of adiponectin has been accounted for by increases in the expression of adiponectin in the adipose tissue of adipocyte-specific insulin receptor-deficient mice (34). Of note, blood adiponectin levels are shown to be elevated even in humans with genetic aberrations in insulin receptors who are associated with severe insulin resistance (35). These findings suggest that insulin stimulation in adipose tissue may lead to inhibition of adiponectin expression. Again, it is shown that decreased SIRT1 signaling, oxidative stress and inflammation lead to decreases in blood adiponectin levels in obesity, in contrast to those seen in starvation and under caloric restriction.

As for the relationship between impaired glucose metabolism and adiponectin, it is shown in a prospective study that the higher the blood adiponectin level, the lower the risk for diabetes in humans (36) and that the blood adiponectin level represents a better marker for the risk of onset of diabetes than blood glucose and insulin levels (37). Again, it has become clear that the greater the HMW adiponectin level, the lower the incidence of diabetes (38). Furthermore, as regards the relationship between dyslipidemia and adiponectin, research suggests a positive correlation between blood adiponectin levels and HDL-cholesterol levels, as well as a negative correlation between blood adiponectin levels and triglyceride levels in humans (39).

Thus, while adiponectin may with its systemic effects on glucose and lipid metabolism indirectly inhibit atherosclerosis, it also acts on vascular endothelial cells and inflammatory cells thus directly inhibiting atherosclerosis in humans. Of note, it is shown that vascular damage is associated with worsening of neointimal formation in adiponectin-deficient mice (20, 40), while adiponectin overexpression is shown to lead to inhibition of atherosclerosis in ApoE-deficient mice, which are known to serve as a mouse model of atherosclerosis (22). Again, it is shown that, even after adjustment for other risk factors, high blood adiponectin levels are associated with significant reductions in the risk of new onset of myocardial infarction in humans, with reports also available to demonstrate the effects of adiponectin on cardiovascular disease (41–43). It is also known that ischemia reperfusion injury increases myocardial apoptosis and the expression of TNF-α in adiponectin-deficient mice leading to increases in myocardial infarct size, suggesting that adiponectin provides direct cardio-protective effects (44).

## Adiponectin Receptors and Their Functions

With a focus on adiponectin binding, our research has led to the successful identification of adiponectin receptors (AdipoR1 and AdipoR2) (45), which are shown to have 66.7% amino-acid homology, with this homology shown to be maintained from yeast to humans, where, of note, the yeast homolog YOL002c is found to have a pivotal role in the oxidation of fatty acids (46). Of these, AdipoR1 is found to be fairly ubiquitously expressed in all tissues but abundantly expressed in skeletal muscle, while AdipoR2 is found to be expressed abundantly in the liver. One of the key features of AdipoRs is that they are novel seven-transmembrane receptors and that their topology is opposite to that of G-protein-coupled receptors (GPCRs) with an intracellular amino-terminus and an extracellular carboxyl-terminus. Again, the presence of AdipoR1 and AdipoR2 were confirmed in siRNA studies to be essential for the binding of adiponectin to the cell membrane surface in cultured cells (45), with loss of their binding and action shown in AdipoR1/AdipoR2 double-knockout mice, demonstrating that AdipoRs represent essential adiponectin receptors in the body (47).

Again, while AdipoR1/AdipoR2 double-knockout mice were found to be associated with insulin resistance and impaired glucose tolerance, this was accounted for by increased inflammation and oxidative stress in major metabolic organs, such as the skeletal muscle, liver and adipose tissue, leading to increased gluconeogenesis and decreased glucose uptake in these mice (47). Furthermore, the expression of AdipoR1 and AdipoR2 was found to be decreased in a mouse model of obesity/type 2 diabetes, which likely accounted in part for the onset of diabetes in the model, while adenovirus-mediated restoration of AdipoR1 expression in the liver was found to activate the AMPK pathway and restoration of AdipoR2 expression was found to activate PPARα, promote fatty acid oxidation, and improve impaired glucose tolerance *in vivo* through its anti-oxidative stress effects (47).

As for adiponectin binding proteins, it is shown that the both calreticulin and CD91 are involved in adiponectin-stimulated uptake of apoptotic cells (48), and pretreatment with anti-calreticulin antibodies attenuates the protective effects of adiponectin on myocyte apoptosis (49). It is also shown that T-cadherin is a receptor for hexameric and HMW adiponectin (50). Moreover, it is reported that in T-cadherin-knockout mice, adiponectin fails to act on cardiac tissue, and circulating levels of adiponectin are dramatically elevated (51, 52). Interestingly, T-cadherin is essential for adiponectin-mediated cardioprotection in mice (51). However, T-cadherin is thought to have no effect on adiponectin signaling, because T-cadherin has no intracellular domain. Further research, including studies with AdipoR1 and/or AdipoR2-knockout mice is necessary to elucidate the molecular mechanism by which adiponectin exerts its cardioprotective effects.

## Transcriptional Regulation of AdipoR Expression in Physiological and Obese Conditions

**Figure 1:**
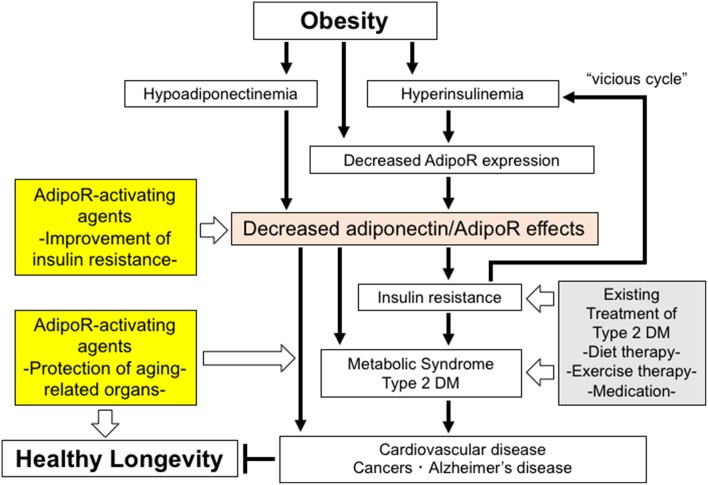
The roles of decreased adiponectin/AdipoR effects in obesity-related diseases and potential strategies to increase the effects of AdipoR. Decreased adiponectin/AdipoR effects play causal roles in obesity-related diseases. Increased activation of AdipoR pathways may have beneficial effects on healthy longevity and obesity-related diseases, such as metabolic syndrome, type 2 diabetes, cardiovascular diseases, cancers, and Alzheimer's disease.

In target organs for insulin, e.g., liver and skeletal muscle, the expression of AdipoR1 and AdipoR2 is shown to significantly increase in fasted mice and decrease in refed mice. Consistently, *in vitro* studies show that insulin leads to reductions in the expression of AdipoR1 and AdipoR2 via the phosphoinositide 3 kinase/FoxO1-dependent pathway. It is shown that the expression of both AdipoR1/R2 is significantly decreased in the adipose tissue and muscle of insulin-resistant ob/ob mice, probably due in part to obesity-associated hyperinsulinemia through FoxO (53), with adiponectin-induced AMPK activation also shown to be impaired in the skeletal muscle of these mice, suggesting that adiponectin resistance is present in ob/ob mice, clearly due to decreases in the expression of AdipoR1/R2. Therefore, obesity is thought to lead to decreases not only in plasma adiponectin levels but also in AdipoR1/R2 expression, thereby reducing adiponectin sensitivity and inducing insulin resistance, which, in turn, leads to hyperinsulinemia thus resulting in a “vicious cycle” (53) (Figure 1). It is also reported that the expression of the AdipoRs is decreased in the skeletal muscle of type 2 diabetic patients (54). Furthermore, it is shown that the expression of the AdipoR gene is correlated with insulin sensitivity in non-diabetic Mexican Americans with or without a family history of type 2 diabetes (55).

## Signal Transduction Mechanisms

The total activity of PGC-1α was found to be decreased in skeletal muscle-specific AdipoR1-deficient mice to one-fourth that seen in normal mice, with decreases also found in mitochondrial mass and function and in proportion of type I muscle fibers, which led not only to decreased exercise endurance but to impaired glucose tolerance and insulin resistance at the organism-level (56). It was also found that adiponectin/AdipoR1 increases intracellular calcium (Ca^2+^) and AMP concentrations, thus offering exercise-mimicking signaling. One major finding of interest is that Ca^2+^ signaling and AMPK/SIRT1 activity were only partially restored, even with a range of agents targeting these pathways, in the phenotype represented by skeletal muscle-specific AdipoR1-deficient mice, suggesting the need for such concurrent activation of Ca^2+^ signaling and the AMPK/SIRT1 pathways as seen with exercise (56).

Although it is shown that AdipoR1 and AdipoR2 are involved in the regulation of glucose and fatty acid metabolism mainly through the activation of Ca^2+^ signaling and the AMPK/SIRT1, and PPARα signaling pathways, it appears likely that additional signaling pathways are also implicated in the pleiotropic actions of adiponectin. Indeed, research suggests that ceramide signaling may also be involved as an additional pathway in mediating such pleiotropic effects (57) and that adiponectin reduces cellular ceramide levels through the activation of ceramidase, as it induces conversion of ceramide to sphingosine, thus leading to reductions in hepatic ceramide levels and improvements in insulin sensitivity.

Conversely, adiponectin deficiency is shown to induce increases in hepatic ceramide levels, which may account in part for insulin resistance, while adiponectin is shown to increase sphingosine 1-phosphate (S1P) thus protecting against apoptosis machinery, such as induced by C2-ceramide or palmitate in pancreatic β cells and cardiac myocytes (57). Given that this protective effect is reversed by S1P itself or a ceramide biosynthesis inhibitor, it is likely that adiponectin-induced S1P protects β cells and cardiac myocytes from apoptotic cell death. Most importantly, this ceramide pathway, which is thought likely to be activated by adiponectin, is totally dependent on the expression of AdipoR1 and AdipoR2, given the observation that the overexpression of adiponectin, AdipoR1, and AdipoR2 leads to reductions in hepatic ceramide levels, as well as improvements in insulin sensitivity.

## AdipoR Agonists in Development

Changes produced in the metabolic capacity of individual organisms by enhanced adiponectin/AdipoR signaling are expected to contribute to the maintenance of their homeostasis. Thus, expectations are mounting for adiponectin/AdipoR-enhancing and AdipoR-activating agents, which appear to have great promise as exercise-mimicking agents providing similar effects to those with exercise, thus opening a new avenue not only for definitive therapy for the diabetes/metabolic syndrome and atherosclerosis but for effective therapeutic options for those unable to exercise due to medical or locomotive conditions. Against this background, our research has led to the successful identification of a small-molecule AdipoR-activating compound (adiponectin receptor agonist; AdipoRon) through scrutiny of candidate compounds available in the University of Tokyo Drug Discovery Innovation Center compound library (58). To date, it is shown that AdipoRon not only improves metabolism at the organ level (e.g., skeletal muscle, liver, and adipose tissue) but provides anti-diabetic properties at the organism level, while it normalizes a shortened lifespan due to obesity (58) (Figure 1).

## Crystal Structures of AdipoR1 and AdipoR2

AdipoR1 and AdipoR2 have been found to have novel structures, with each containing an internal cavity inside the seven transmembrane helices coordinating a zinc ion, demonstrating that their functions and structures are distinct from those of conventional GPCRs (59, 60).

A recent research showed further insights into the AdipoR1/AdipoR2 functions and structures (61), showing that they possess enzymatic activity, despite their extremely low ceramidase activity. Therefore, further research is required to characterize the parameters for their enzymatic activity and their substrate specificity, as well as to identify their further functions that may remain to be unraveled.

## Conclusions

Ever since the elucidation in 2007 of the structure of a first human GPCR, GPCR research is being increasingly accelerated, with expectations mounting for further findings from their structural analysis that may also offer important leads for drug discovery. It is hoped that further structural analysis of AdipoRs will lead not only to elucidation of the mechanisms of signal transduction involved in AdipoRs as novel seven-transmembrane receptors but to provide invaluable clues for optimization of the adiponectin receptor agonist AdipoRon for human use (best-in-class). In conclusion, adiponectin receptor-activating agents appear to hold great promise as effective options for the prevention and treatment of type 2 diabetes and atherosclerosis, thereby contributing to healthy longevity in humans (62–64).

## Author Contributions

MI, MO-I, TY, and TK wrote the manuscript. All authors reviewed the manuscript.

### Conflict of Interest Statement

The authors declare that the research was conducted in the absence of any commercial or financial relationships that could be construed as a potential conflict of interest.
